# Assessing anesthetic activity through modulation of the membrane dipole potential

**DOI:** 10.1194/jlr.M073932

**Published:** 2017-08-17

**Authors:** Benjamin Michael Davis, Jonathan Brenton, Sterenn Davis, Ehtesham Shamsher, Claudia Sisa, Ljuban Grgic, M. Francesca Cordeiro

**Affiliations:** University College London Institute of Ophthalmology,* London EC1V 9EL, United Kingdom; Western Eye Hospital,† Imperial College Healthcare National Health Service Trust, and Imperial College Ophthalmic Research Group, Imperial College London, London NW1 5QH, United Kingdom

**Keywords:** cholesterol, diagnostic tools, eye, lipid rafts, model membranes, accidental awareness during general anesthesia, retina, di-8-ANEPPs, fluorescence spectroscopy

## Abstract

There is great individual variation in response to general anesthetics (GAs) leading to difficulties in optimal dosing and sometimes even accidental awareness during general anesthesia (AAGA). AAGA is a rare, but potentially devastating, complication affecting between 0.1% and 2% of patients undergoing surgery. The development of novel personalized screening techniques to accurately predict a patient’s response to GAs and the risk of AAGA remains an unmet clinical need. In the present study, we demonstrate the principle of using a fluorescent reporter of the membrane dipole potential, di-8-ANEPPs, as a novel method to monitor anesthetic activity using a well-described inducer/noninducer pair. The membrane dipole potential has previously been suggested to contribute a novel mechanism of anesthetic action. We show that the fluorescence ratio of di-8-ANEPPs changed in response to physiological concentrations of the anesthetic, 1-chloro-1,2,2-trifluorocyclobutane (F_3_), but not the structurally similar noninducer, 1,2-dichlorohexafluorocyclobutane (F_6_), to artificial membranes and in vitro retinal cell systems. Modulation of the membrane dipole provides an explanation to overcome the limitations associated with the alternative membrane-mediated mechanisms of GA action. Furthermore, by combining this technique with noninvasive retinal imaging technologies, we propose that this technique could provide a novel and noninvasive technique to monitor GA susceptibility and identify patients at risk of AAGA.

General anesthetics (GAs) are potent and reversible inducers of muscle relaxation, analgesia, immobility, reflex suppression, and sleep, without which many aspects of modern medicine would be impossible. For this reason, GAs are widely considered to be one of the most important medical advances in the last 200 years. However, the mechanism of action of this diverse family of molecules remains poorly understood (1), as is the individual variation in patient response. This is an important problem, as a complete understanding of this mechanism will permit the design of molecules with improved safety and activity, and permit more accurate predictions of an individual’s response to GA. Such an advance would contribute to developing better techniques to assess adequate levels of GA to administer and provide a personalized medicine approach to rapidly determine the best combination of GA agents for each patient. Furthermore, there is a risk of rare, but significant, complications presently associated with anesthetic administration, including malignant hyperthermia (2), succinylcholine-related apnea (3), anaphylaxis (4), accidental awareness during general anesthesia (AAGA) (5), and GA-associated mortality (6, 7).

AAGA is defined as the recall of events or experiences that occurred during anesthesia and is a potentially devastating complication affecting between 0.1% and 2% of all patients undergoing GA (8–10). Over three-quarters of the cases of AAGA are caused by a period of awareness under GA of less than 5 min duration. However, long-term adverse effects affect 41% of people with this condition (11). Patient recollection of AAGA range widely from nondistressing audiological recall to extremely distressing recollection of agony and paralysis, often associated with the development of posttraumatic stress disorder-like symptoms (10, 12). Multiple potential risk factors for AAGA have been identified by the recent NAP5 study, including the type of surgery (obstetric, cardiac, and pediatric), higher patient American Society of Anesthesiologists score, and obesity (13). In addition, deficiencies in labeling and vigilance are also reported to contribute to AAGA (14) and an intrinsic (possibly genetic) resistance to GA has been suggested, with between 1.6% and 11% of patients reporting a previous history of AAGA (15, 16).

There is growing evidence to suggest that better monitoring of the depth of anesthesia can reduce the risk of AAGA (17). Depth of anesthesia is routinely monitored using the isolated forearm technique to measure the responsiveness to command as a consciousness surrogate, and quantifying the end-tidal concentration of volatile anesthetics or plasma levels of intravenously administered agents to assess anesthetic delivery (9). Each of these techniques, however, is of limited clinical utility, as only 50% of patients who respond to command with an isolated forearm can later recall doing so (9) and monitoring the extent of anesthetic delivery is not the same as monitoring the actual effectiveness of the GA (8). Alternative methods of monitoring response to anesthesia and measuring the level of consciousness include recording the electrical activity of the brain with evoked potentials and electroencephalograms. Despite the availability of these techniques for over 20 years (18), brain monitoring techniques are not routinely used in clinical practice. This is due to the difficulty in distinguishing brain activity from internal (muscular) and external (surgical device) interference, and different excitatory and inhibitory effects of GAs on different ion channels giving rise to complex electroencephalogram responses that can be further complicated by administration of non-GA drugs (8). As such, brain monitoring techniques are presently considered only qualitative indicators of GA depth (9, 19), meaning that there is a clear unmet need to develop techniques to accurately and quantitatively predict a patient’s response to anesthesia.

The membrane dipole potential describes an electrical potential that arises from the organization of dipoles within and on the membrane surface [for a comprehensive review see (20)]. Ordinarily, the membrane dipole potential has a magnitude of between 200 and 400 mV, depending on membrane composition (21), and is independent of membrane fluidity (22). The dipole potential is also sensitive to cholesterol content (23) and stereospecificity (24) and can be used to modulate the activity of the raft-associated (25) membrane protein, P-glycoprotein (26–28). The theory of GA-mediated modulation of membrane dipole potential was first proposed by Qin, Szabo, and Cafiso (29). The authors reported that addition of 1 minimum alveoli concentration (MAC) equivalent concentrations of the GAs, enflurane, isoflurane, and halothane, to artificial membranes induced substantial reductions in the membrane dipole potential (−10.5, −10.5, and −6.7 mV, respectively). Other groups have also demonstrated that modulation of the membrane dipole potential can affect gramicidin A channel activity (30) [more recently with local anesthetics (31)] and sodium potassium pumps (32). Together, these data suggest that modulation of the membrane dipole potential could provide an attractive mechanism of indirect GA-induced modulation of membrane protein activity without requiring direct protein-ligand interactions.

The retina comprises the only portion of the CNS that can be readily visualized at the cellular level using noninvasive imaging techniques, such as confocal scanning laser ophthalmoscopy (33). Changes in retinal function have been reported using multifocal electroretinography, where isoflurane anesthesia induction in a porcine model resulted in a reduced RGC response (34). Building on this observation, we sought to determine the feasibility of monitoring retinal neuronal cell behavior in response to GA induction and whether this technique could ultimately be used to provide a screening tool to rapidly and quantitatively assess a patient’s individual response to anesthesia prior to undergoing surgery. In the present study, we sought to determine whether the small molecule ratiometric probe, di-8-ANEPPs, which becomes strongly fluorescent only when incorporated into the cell membrane and has previously been used extensively to report on the membrane dipole potential (27, 28, 35), can be used to specifically report on anesthesia response using in vitro models. This was achieved by exposing di-8-ANEPPs-labeled artificial membrane (liposomes) and retinal neuronal cell lines to varying concentrations of either 1-chloro-1,2,2-trifluorocyclobutane (F_3_) or 1,2-dichlorohexafluorocyclobutane (F_6_), an anesthetic/nonanesthetic pair with high structural similarity (**Fig. 1**) (36, 37). F_3_ has been previously reported to inhibit presynaptic voltage-gated sodium channels and potentiate GABA_A_ chloride channels, while F_6_ has been found to have no effect on either channel (38, 39). The di-8-ANEPPs was chosen for this purpose, as methods to convert the fluorescence ratio measured to quantitative membrane dipole potential changes are established (23). The ratiometric nature of di-8-ANEPPs also ensures that this measurement is independent of changes in partitioning of the probe between the membrane and aqueous milieu. The use of this probe with microscopic imaging techniques has been reported (40) and it is increasingly being used for in vivo applications (41). A proposed schematic for such a screening device for use in preoperative conscious patients measuring their unique responses to subclinical doses of GA or GA combinations is discussed.

**Fig. 1.**
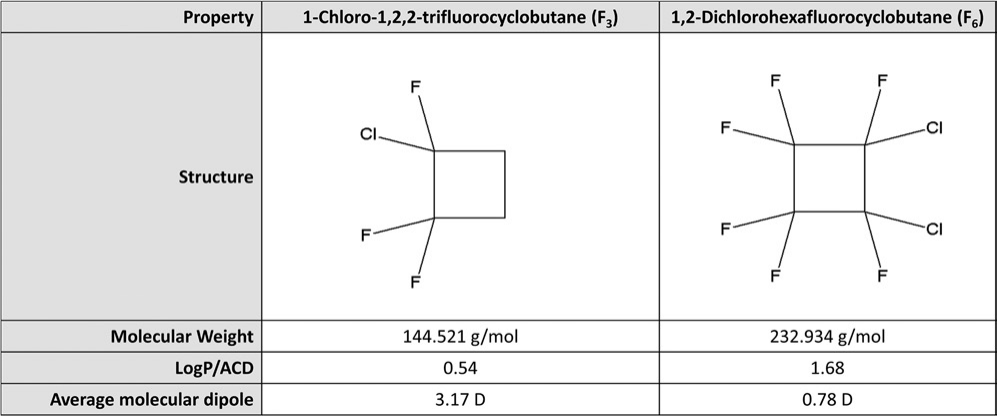
Properties of the volatile anesthetic (F_3_) and the nonimmobilizer (F_6_). F_3_ and F_6_ possess similar lipophilicity [calculated using ACD/LogP (octanol/water partition coefficient) 12.0], but distinct average molecular dipoles (36), as a result of the more unequal distribution of electronegative halogen atoms in F_3_ than in F_6_.

## METHODS

### Reagents

All materials and reagents were acquired at the highest purity from Sigma-Aldrich (Kent, UK) unless otherwise stated. All statistical analyses were carried out using GraphPad Prism version 5.00 for Windows (GraphPad Software, San Diego, CA).

### Unilamellar liposome preparation

Egg phosphatidylcholine (PC) and cholesterol (ovine) were purchased from Avanti Polar Lipids (Alabaster, AL). Liposomes were prepared using a lipid film hydration technique (42). Briefly, 13 mM of PC_100%_ or PC_70%_Cholesterol_30%_ (molar ratios) were dissolved in a chloroform:methanol (5:1 ratio) solvent and dried under reduced pressure (50 mBar, 1 h at 45°C) to form a thin film. This film was rehydrated in sucrose-Tris buffer [280 mM sucrose, 10 mM Trizma-hydrochloride (pH 7.4), at 45°C for 1 h with agitation. The resulting liposome suspension was extruded sequentially through polycarbonate filters with pores of 400, 200, and 100 nm in diameter (Nucleopore Corp., Pleasanton, CA) using a handheld extruder (Mini-extruder; Avanti Polar Lipids) to produce a solution of unilamellar phospholipid vesicles of uniform size. Liposomes were labeled exclusively in the outer bilayer leaflet with di-8-ANEPPs, as described previously (27). Briefly, liposomes were incubated for 1.5 h at 37°C in the presence of di-8-ANEPPs (Thermo Fisher Scientific, Waltham, MA) dissolved in ethanol while protecting from light.

### Liposome characterization by dynamic light scattering

Liposome size was determined using a Zetasizer Nano-ZS and Zetasizer 7.02 software (Malvern Instruments, Malvern, UK). Liposome suspensions were diluted to a concentration of 400 μM in sucrose-Tris buffer [280 mM sucrose, 10 mM Trizma-hydrochloride (pH 7.4), and particle size recorded prior to and following 5 mM additions of F_3_ or F_6_ using dynamic light scattering. The particle diameter (Z-average) and polydispersity of the liposome population were recorded for three populations, from which the average particle diameter was calculated.

### Immortalized retinal neuronal cell culture

An immortalized retinal neuronal cell line (a gift from Dr. Neeraj Agarwal, Department of Cell Biology and Genetics, University of North Texas Health Science Center, Fort Worth, TX) was used in this study as a model neuronal cell. These neurons express the typical retinal and neuronal markers, Thy-1, Brn-3a, and β3 tubulin (43–45), and are also reported to have similarities to the 661W photoreceptor cell line (46–48). Retinal neurons were cultured in DMEM (Invitrogen, Paisley, UK) supplemented with 10% heat-inactivated fetal bovine serum (Invitrogen). Penicillin (100 U/ml) and streptomycin (100 mg/ml) were used to maintain the cells prior to experimentation.

### The di-8-ANEPPs labeling of neuronal cell suspensions

Cell suspensions were counted using a trypan blue exclusion assay before harvesting by centrifugation (300 *g*, 5 min). Cells were labeled with di-8-ANEPPs according to the methods outlined by Asawakarn, Cladera, and O’Shea (28). Briefly, 10 μM of di-8-ANEPPs were added to a suspension of cells (1 × 10^6^ cells·ml^−1^ sucrose-Tris buffer) for 1.5 h at 37°C with mixing. Labeled cells were used for experiments within 2 h of labeling. When required, depletion of cholesterol from cell membranes was achieved by treating di-8-ANEPPs cell suspensions for 1 min with 10 mM methyl-β-cyclodextrin (MβCD) before removal by centrifugation (300 *g* for 5 min at 25°C) and resuspension in fresh sucrose buffer. Cells were used for experiments within 1 h of cholesterol depletion.

### Spectral and ratiometric fluorescent recordings

Fluorescence measurements were taken during the addition of the desired amounts of F_3_, F_6_, or primary alcohols to suspensions of liposomes or cells (400 μM lipid or 40,000 cells·ml^−1^ sucrose-Tris buffer). Measurements were acquired on a Cary Eclipse fluorescence spectrophotometer (Agilent Technologies) and during acquisition samples were maintained at 37°C with magnetic stirring. Fluorescence difference spectra were obtained by subtracting the di-8-ANEPPs excitation spectra after the addition of agents of interest from those obtained at baseline. Before subtraction, each spectrum was normalized to the integrated areas so that the difference spectra would reflect only the spectral shifts (28). Each difference spectrum was then normalized to an appropriate buffer control and a three-point moving average applied to reduce noise.

The di-8-ANEPPs ratiometric time series were obtained using excitation wavelengths of 420 and 520 nm and an emission wavelength of 670 nm. Additions of 1, 2.5, or 5 mM of F_3_ or F_6_ were made after approximately 120 s of baseline recording and acquisition was continued until no further change in di-8-ANEPPs fluorescence signal was observed (typically between 300 and 600 s). Data were fit using linear regression (indicative of no significant change in di-8-ANEPPs ratio on addition of ligands) or plateau followed one-phase exponential decay function equation 1:

(Eq. 1) $$\text{Y}=\text{IF}\left\{\text{X}<{\text{X}}_{0},{\text{Y}}_{0},\text{Plateau}+\left({\text{Y}}_{0}-\text{Plateau}\right)\times \exp\left[-\text{K}\times \left(\text{X}-{\text{X}}_{0}\right)\right]\right\}$$

where X_0_ is the time at which the decay begins (fixed as the time of GA/non-GA addition), Y_0_ is the average Y value at time X_0_, plateau is the value of Y at infinite time, and K is the rate constant expressed as s^−1^. The preferred model in each case was determined using an extra sum-of-squares F-test and the simpler model (linear regression) was selected, unless *P* < 0.05. The di-8-ANEPPs fluorescence ratios were converted into estimates of the membrane dipole potential using the calibration equation described by Starke-Peterkovic (23) (equation 2):

(Eq. 2) $$\psi_{\text{d}}\;=\frac{\text{R}+\text{a}}{\text{b}}$$

where ψ_d_ is the membrane dipole potential, R is the ratiometric intensity of di-8-ANEPPs fluorescence ratio, a = 0.3 (±0.4), and b = 4.3 (±1.2) × 10^−3^ mV^−1^. In agreement with previous studies, as the large errors associated with a and b are thought to arise from difficulties in determining the absolute value of ψ_d_ and we were principally concerned with relative changes in ψ_d_, only the variance in R was considered in error calculations (40).

### Membrane fluidity measurements

Liposomes were labeled with the fluorescent probe, 9-(dicyanovinyl)julolidine (DCVJ), as described previously (49). Briefly, 13 mM PC_100%_ and PC_70%_Cholesterol_30%_ liposomes were labeled with 45 μM DCVJ for 1.5 h at 37°C. Liposomes were diluted to 400 μM (1.5 μM DCVJ) with HEPES saline buffer [10 mM HEPES, 140 mM sodium chloride (pH 7.4)]. Fluorescence emission (490 nm) was recorded at an excitation of 430 nm during the addition of DMSO, F_3_, and F_6_. Measurements were acquired using a Cary Eclipse fluorescence spectrophotometer (Agilent Technologies) and during acquisition, samples were maintained at 37°C with magnetic stirring. Under the assumption of constant temperature and absorption, change in membrane fluidity (η_1_/η_2_) can be determined from DCVJ fluorescence emission on addition of DMSO, F_3,_ or F_6_ using equation 3 (50);

(Eq. 3) $$\frac{\eta_{1}}{\eta_{2}}=\;{\left(\frac{{\text{I}}_{1}-{\text{I}}_{\text{o}}}{{\text{I}}_{2}-{\text{I}}_{\text{o}}}\right)}^{\frac{1}{\text{x}}}$$

where I_1_ and I_2_ are the fluorescence emission from DCVJ before and after addition of an agent that modulates membrane fluidity, x is a constant equal to 0.6 for DCVJ, and I_o_ is the signal due to nonfluorescent scattering, filter bleed-through, and ambient light effects (here I_o_ = 110.23).

### Cell viability

An AlamarBlue resazurin reduction assay (Invitrogen) was conducted, as per the manufacturer’s instructions, to measure the potential toxicity of all concentrations of anesthetic molecules used. Cells were incubated with the anesthetic compounds for 2 h at 37°C with 5% CO_2_ and, following incubation, 10% AlamarBlue was added. A cell-free control containing 10% AlamarBlue only was included as a negative control. Fluorescence was measured on a Safire plate reader (Tecan, Zurich, Switzerland) with an excitation wavelength of 570 nm and an emission wavelength of 585 nm. Percentage viability was determined by normalization to untreated (100% viable) and cell-free control (0% viable) groups.

## RESULTS

### Addition of F_3_, but not F_6_, to liposomes induced a dose-dependent reduction in membrane dipole potential without membrane aggregation

**Fig. 2.**
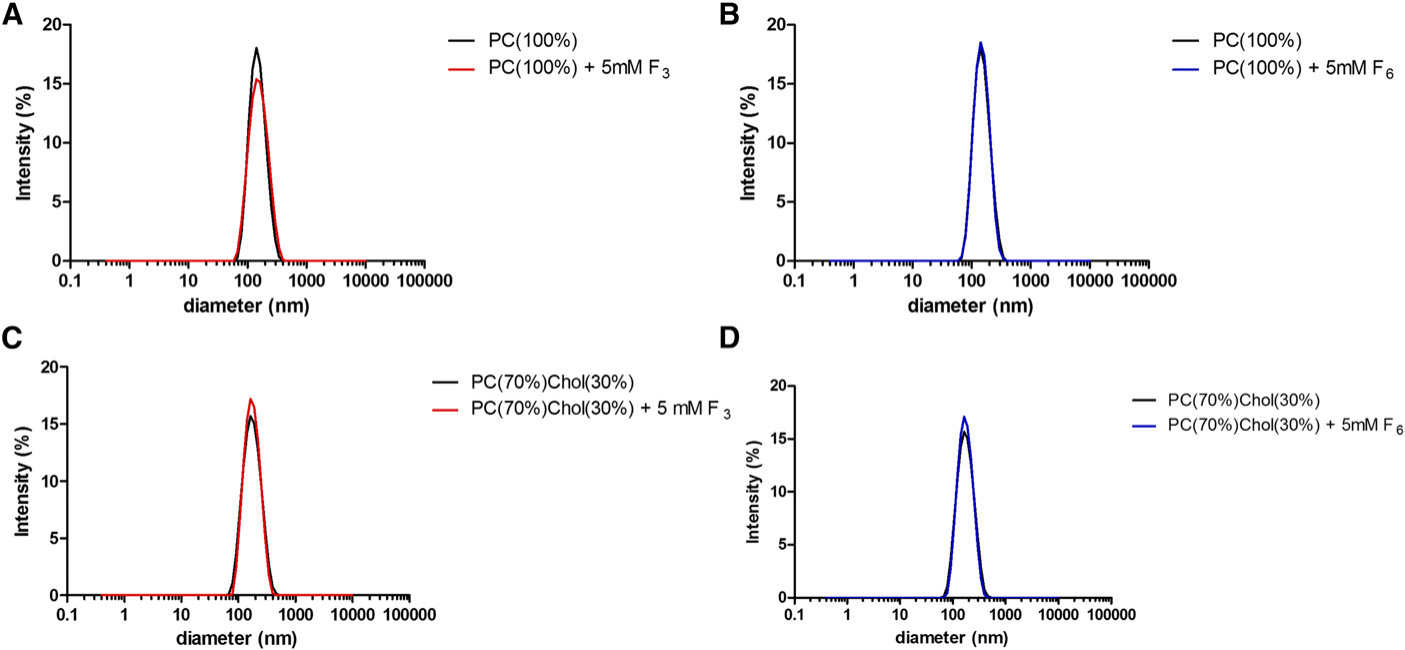
Addition of 5 mM F_3_ or F_6_ did not induce liposome aggregation. Dynamic light scattering demonstrates that addition of 5 mM F_3_ (A, C) or F_6_ (B, D) did not induce significant aggregation of PC_100%_ (A, B) and PC_70%_Cholesterol_30%_ (C, D) liposomes after incubation for 30 min at 37°C (n = 3).

**Fig. 3.**
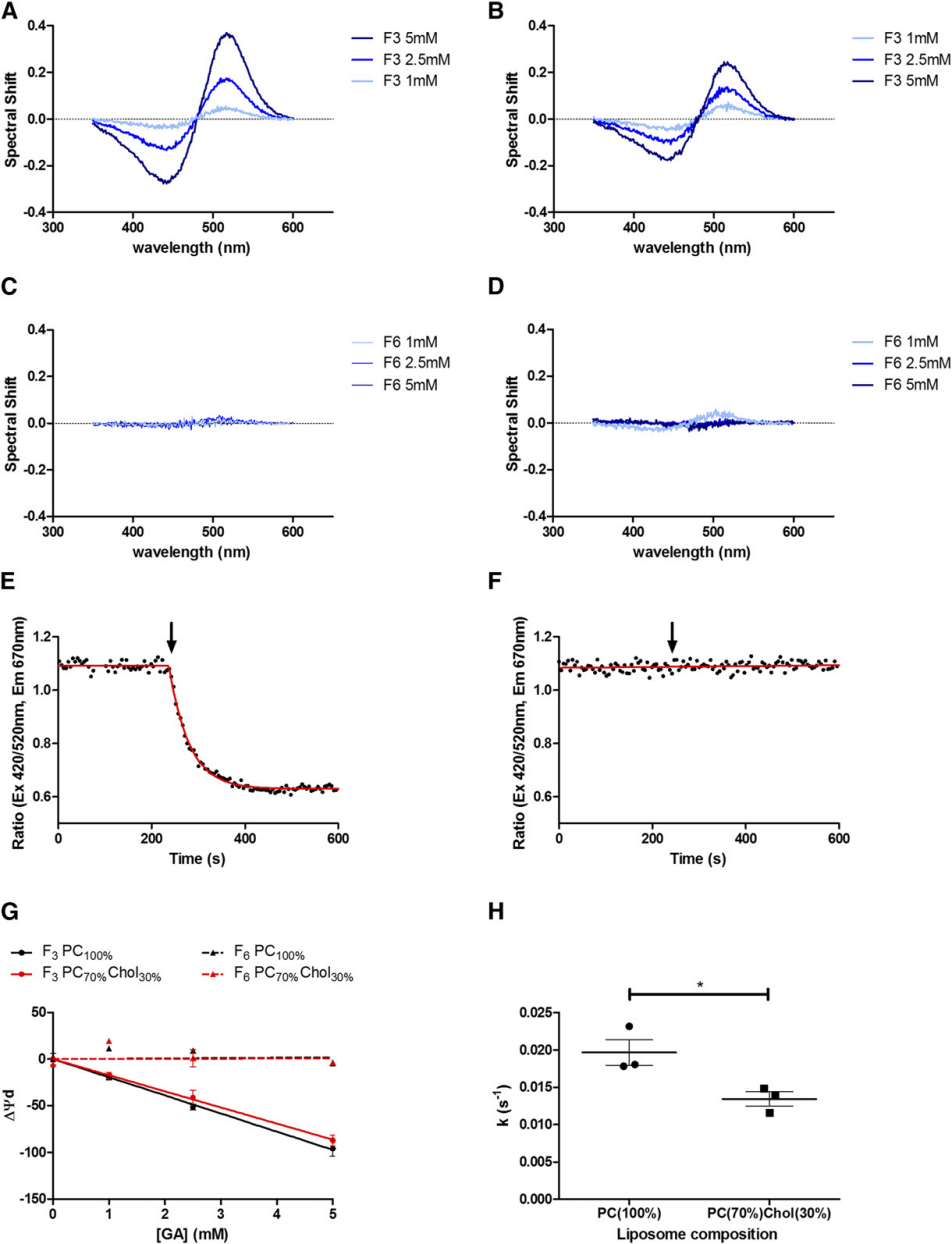
Comparing the interactions of F_3_ and F_6_ with artificial membrane systems and their effects on the membrane dipole potential. A–D: The fluorescence excitation spectra of di-8-ANEPPs-labeled liposomes shifts in response to F_3_, but not F_6_, indicative of a reduction in the membrane dipole potential. Fluorescence difference spectra obtained by subtracting excitation spectra (λ_em_ = 670 nm) of di-8-ANEPPs-labeled PC_100%_ [PC(100%)] (A, C) and PC_70%_Cholesterol_30%_ [PC(70%)Chol(30%)] (B, D) liposomes (400 μM) from those obtained on addition of the indicated concentrations of F_3_ (A, B) or F_6_ (C, D). Before subtraction, each spectrum was normalized to their integrated areas so that the difference spectra only reflected the spectral shift. In each experiment, temperature was maintained at 37°C. A dual wavelength ratiometric measurement of the membrane dipole potential was made on addition of F_3_ (E) or F_6_ (F) to di-8-ANEPPs-labeled PC_100%_ liposomes at the indicated time (arrows represent 5 mM additions). Samples were excited at 420 and 520 nm and emission recorded at 670 nm. The ratio R(420/520) was calculated (black dots) before each titration was fit (red line) to a plateau followed by one-phase decay (equation 1) or straight line (equation 2) using an extra sum-of-squares F-test to determine the best fitting model in each case. G: Addition of F_3_ (solid lines), but not F_6_ (dashed lines), to PC_100%_ (black) and PC_70%_Cholesterol_30%_ (red) artificial membrane systems induced a dose-dependent reduction in the membrane dipole potential. All experiments n = 3; mean ± SEM. H: The rate of change in membrane dipole potential on addition of 5 mM F_3_ to PC_100%_ liposomes was significantly greater (two tailed unpaired *t*-test, *P = 0.0352).

Addition of 5 mM F_3_ or F_6_ to 400 μM PC_100%_ or PC_70%_Cholesterol_30%_ liposomes did not induce significant liposome aggregation, retaining comparable average population diameters and a polydispersity index of <0.2 in each case (**Table 1** and **Fig. 2**). Titration of 1, 2.5, and 5 mM con­centrations of F_3_ into di-8-ANEPPs-labeled PC_100%_ and PC_70%_Cholesterol_30%_ liposomes caused a concentration-dependent red-shift in the excitation spectrum (**Fig. 3A**, B), indicative of a reduction in the membrane dipole potential (27). The magnitude of the F_3_-induced red-shift in the di-8-ANEPPs excitation spectra was reduced in PC_70%_Cholesterol_70%_ versus PC_100%_ membranes, indicative of a smaller change in the membrane dipole potential in this membrane system. In contrast, no detectable spectral shift was recorded when liposomes were titrated with equivalent concentrations of the nonimmobilizer, F_6_ (Fig. 3C, D).

**TABLE 1.** Characteristics of liposomes in the presence or absence of F_3_ and F_6_

	PC_100%_			PC_70%_Cholesterol_30%_		
	Naïve	Plus 5 mM F_3_	Plus 5 mM F_6_	Naïve	Plus 5 mM F_3_	Plus 5 mM F_6_
Z-average diameter (nm ± SD)	142 (48)	144 (57)	141 (46)	163 (64)	164 (57)	163 (57)
Polydispersity index	0.099	0.110	0.060	0.105	0.088	0.084

Recording changes in di-8-ANEPPs 420/520 nm fluorescence ratio over time confirmed that addition of F_3_ to PC_100%_ liposomes caused a concentration-dependent decrease in this parameter (Fig. 3D), while addition of equivalent concentrations of F_6_ did not cause any apparent change (Fig. 3E). Titration of F_3_ fit best to a plateau followed by single exponential decay function equation 1, while those involving F_6_ typically best fit to a straight line function (indicative of no significant change in dipole potential) or exponential decay function with nominal span. Figure 3F illustrates that the change in dipole potential on addition of F_3_ declined significantly and linearly with increasing concentration (F-test *P* < 0.0001 for PC_100%_- and PC_70%_Cholesterol_30%_-containing liposomes), while the addition of F_6_ had no such relationship (slope ± 95% CI; PC_100%_ 0.39 ± 4.06 mV/mM F_6_ and PC_70%_Cholesterol_30%_ 0.15 ± 6.33 mV/mM F_6_, F-test, *P* = 0.82 and *P* = 0.95, respectively). On comparing the gradient of each line, addition of F_3_ to cholesterol-containing liposomes was found to induce a smaller change in the membrane dipole potential than addition to those comprised of PC alone (slope ± 95% CI; PC_100%_ −19.45 ± 1.07 mV/mM F_3_ vs. PC_70%_Cholesterol_30%_ −17.26 ± 0.65 mV/mM F_3_). Finally, the rate of dipole potential change on addition of F_3_ to PC_100%_ liposomes was found to be almost twice that found on addition of this GA to PC_70%_Cholesterol_30%_ membranes (Fig. 3G; 0.020 ± 0.003 s^−1^ vs. 0.013 ± 0.002 s^−1^, respectively, two-tailed unpaired *t*-test, *P* = 0.0352).

### Addition of primary alcohols (ethanol to dodecanol), but no tetradodecanol, to liposomes induced a dose-dependent reduction in membrane dipole potential without membrane aggregation

**Fig. 4.**
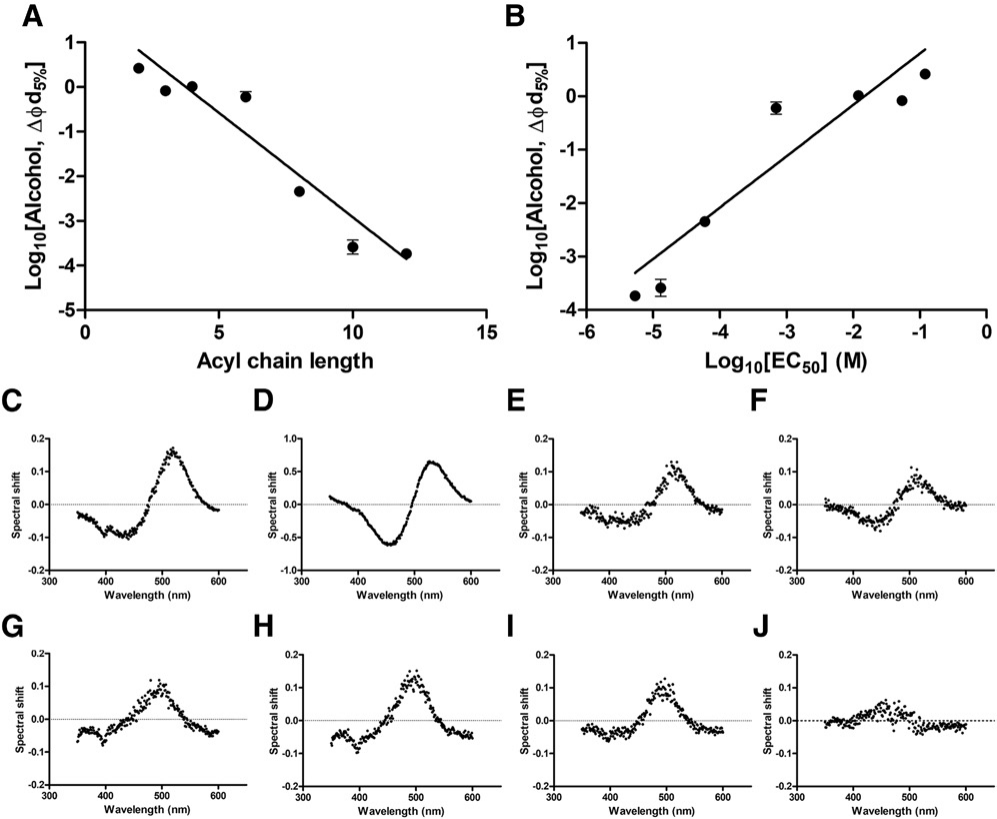
Comparing the interactions of a series of primary alcohols with artificial membrane systems and their effects on the membrane dipole potential. A: The concentration of primary alcohol required to induce a 5% (∼18 mV) reduction in the membrane dipole potential versus acyl chain length indicates a significant negative correlation (slope = −0.48 ± 0.04, Spearman’s *R* = −0.964, *P* = 0.0028) between acyl chain length and dipole potential modulating effect until tetradodecanol (C14), where a clear cut-off point is observed. B: A significant positive correlation (slope = 0.9618 ± 0.09172, Spearman’s *R* = 0.964, *P* = 0.0028) between the effect of primary alcohols on dipole potential modulation and anesthetic potency previously reported in tadpoles (51, 52). C–J: The fluorescence excitation spectra of di-8-ANEPPs-labeled liposomes shifts in response to addition of primary alcohols [ethanol (C), propanol (D), butanol (E), hexanol (8.9 mM) (F), octanol (2.7 mM) (G), decanol (0.26 mM) (H), and dodecanol (0.15 mM) (I)] indicative of a reduction in the membrane dipole potential. Fluorescence difference spectra obtained by subtracting excitation spectra (λ_em_ = 670 nm) of di-8-ANEPPs-labeled PC_100%_ after addition of primary alcohols from baseline values, as previously described in the text. Interestingly, addition of 0.17 mM tetradodecanol to this artificial membrane system caused no such change in the membrane dipole potential (J).

The homologous series of primary alcohols between ethanol and dodecanol have long been established to possess increasing anesthetic potency with acyl chain length; however, between dodecanol and tetradodecanol, a cut-off point exists beyond which no anesthetic activity is observed (51). To investigate this phenomenon, the ability of primary alcohols to modulate the membrane dipole potential in PC_100%_ artificial membranes was investigated (**Fig. 4**). Titration of primary alcohols with an acyl chain length between 2 and 12 carbons was found to induce a dose-dependent reduction in the membrane dipole potential (Fig. 4C–I), with the concentration required to induce a 5% reduction in dipole potential (∼18 mV) reducing with increasing acyl chain length (Fig. 4A). The concentration of primary alcohols required to induce a reduction in membrane dipole potential significantly correlated (Fig. 4B; slope = 0.9618 ± 0.09172, Spearman’s *R* = 0.964, *P* = 0.0028) with the EC_50_ of these molecules previously reported in tadpoles (51, 52). Titration of membranes with tetradodecanol, however, did not induce a significant change in membrane dipole potential up to concentrations of 0.17 mM (Fig. 4J) with addition of greater concentrations of this alcohol, including visible precipitation in the cuvette.

### Addition of F_3_ and F_6_ to DCVJ-labeled artificial membrane systems induces a small, but physiologically irrelevant, increase in membrane fluidity

**Fig. 5.**
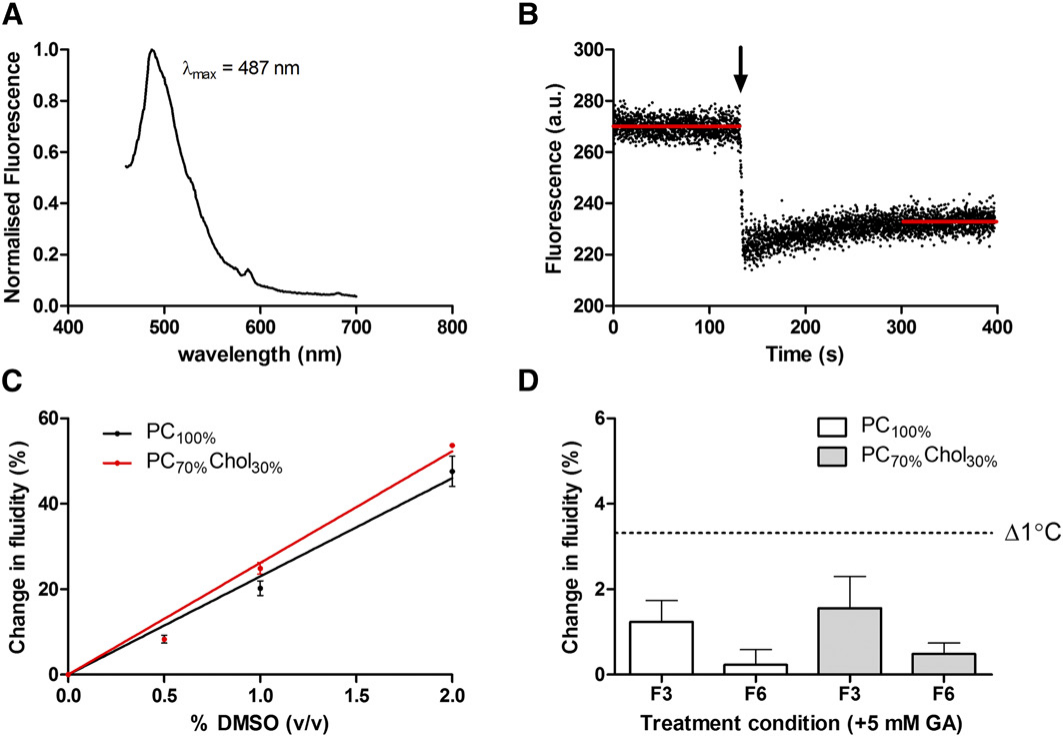
The influence of F_3_ and F_6_ on membrane fluidity. A: Normalized emission spectra of DCVJ-labeled PC_100%_ liposomes (400 μM liposomes with 1.5 μM dye) in HEPES buffered saline (pH 7.4) at 37°C exhibits peak fluorescence emission of 487 nm, similar to that previously reported for PC liposomes (49). B: Addition of 2% DMSO to DCVJ-labeled PC_100%_ liposomes at 37°C induced a reduction in DCVJ fluorescence indicative of an increase in membrane fluidity in agreement with the reported behavior of this molecule in PC- and cholesterol-containing membranes (53, 54). C: A concentration-dependent change in membrane fluidity (DCVJ fluorescence intensity ratio) was observed on addition of DMSO to PC_100%_ and PC_70%_Cholesterol_30%_ [PC_70%_Chol_30%_] liposomes at 37°C. On fitting this data to a linear regression model, DMSO was found to induce a significantly greater change in membrane fluidity in PC_70%_Chol_30%_ membranes, perhaps indicative of disassembly of cholesterol-containing domains (extra sum-of-squares F-test, *P* = 0.0246). D: Addition of F_3_ and F_6_ to liposomes was observed to induce an increase in membrane fluidity with F_3_ modulating the membrane fluidity to a greater extent than F_6_. The extent of membrane fluidity modulation was, however, slight and less than that typically attributed to a change in temperature of 1°C (Table 2). As mammalian brain temperatures are reported to fluctuate under physiological conditions by up to 3°C (93), this strongly suggests that modulation of membrane fluidity is not responsible for GA action.

As the fluorescence emission of DCVJ is dependent on the dielectric constant of its environment (49), the effective labeling of membranes with DCVJ was first assessed by recording the fluorescence emission spectra (**Fig. 5A**). Peak emission wavelength was observed at 487 nm for both membrane compositions, which is similar to that previously reported for PC-containing liposomes (49). A positive control to confirm the efficacy of this technique was the addition of DMSO (0.5% v/v to 2% v/v) to membranes, as it is reported to increase membrane fluidity (53, 54). A dose-dependent increase in membrane fluidity on addition of DMSO was observed (Fig. 5B, C). Addition of 5 mM F_3_ or F_6_ to PC_100%_ or PC_70%_Cholesterol_30%_ liposomes induced a subtle increase in membrane fluidity (Fig. 5D); however, this increase was less than that expected for the change in membrane fluidity that occurs as a result of a change temperature of 1°C (3.32 ± 1.05%, **Table 2**). This parameter was calculated using a previously published dataset (49), where a change in membrane fluidity over a known temperature range for PC-containing liposomes was used to estimate the percentage change in fluidity per 1°C change in temperature from 35°C, not accounting for phase transitions.

**TABLE 2.** The influence of temperature changes on membrane fluidity

Membrane Composition*^a^*	Technique (Probe)*^a^*	Temperature (°C)*^a^*	η (cP)*^a^*	Δη (cP)	Δη (%/°C)*^b^*
DPPC	Quantum yield (DCVJ)	10–60	120–70	50	1.05
DPPC	Quantum yield (DCQEB)	10–60	30–3	27	3.27
DPPC	fluorescence depolarization (perylene)	25–45	940–94	846	8.18
DPPC	fluorescence depolarization (diphenylhexatriene)	10–60	1,000–50	950	3.62
DPPC	intramolecular excimer formation (dipyrenylpropane)	20–50	30–18	12	1.67
DMPC	Intramolecular excimer formation (dipyrenylpropane)	10–60	125–38	87	2.13
Mean (SE)					3.32 (1.05)

a Derived from reference (49).

b Not accounting for phase transitions.

### Addition of F_3,_ but not F_6,_ to immortalized neuronal cells induced a dose-dependent reduction in membrane dipole potential without cell toxicity

**Fig. 6.**
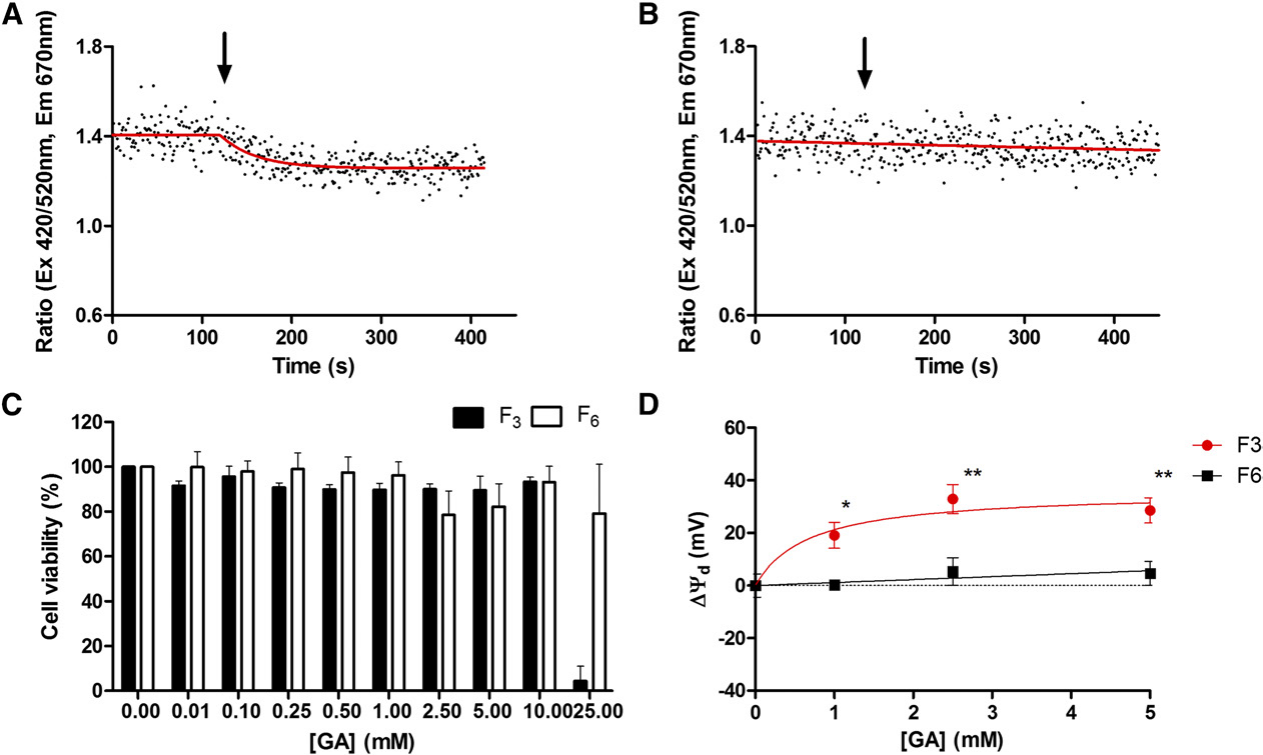
The interaction of F_3_ and F_6_ with neuronal cells. Addition (black arrows) of 5 mM F_3_ (A), but not 5 mM F_6_ (B), induced a reduction in the di-8-ANEPPs fluorescence ratio indicative of a reduction in the membrane dipole potential. C: Immortalized neuronal cell cultures were incubated in the presence of varying concentration of F_3_ or F_6_ for 24 h before cell viability was assessed using an AlamarBlue assay. No significant change in cell viability was observed on addition of up to 10 mM concentrations of either agent. D: The change in membrane dipole potential induced on addition of F_3_ or F_6_ to immortalized neuronal cells labeled with di-8-ANEPPs. Profiles were fit to a simple hyperbolic equation or straight line and the best fitting model determined by F-test, n > 3, mean ± SEM.

Incubation of immortalized neuronal cell lines for 24 h with F_3_ and F_6_ up to concentrations of 10 mM was found to be well-tolerated and cause no significant decline in cell viability versus untreated controls using the AlamarBlue resazurin viability assay (**Fig. 6C**). The di-8-ANEPPs-labeled immortalized neuronal cells had an average basal membrane dipole potential of 367 ± 6 mV. Addition of F_3_ was found to induce a dose-dependent decrease in di-8-ANNEPs fluorescence ratio at 420/520 nm excitation, which fit best to a plateau followed by an exponential decay (Fig. 6A). No detectable change in the di-8-ANEPPs fluorescent ratio was observed on addition of equivalent concentrations of F_6_ to cells (Fig. 6B). Plotting the change in terms of membrane dipole potential revealed that addition of F_6_ to cells up to concentrations of 5 mM induced no significant change in the membrane dipole potential. In contrast, the addition of F_3_ induced a dose-dependent and significant reduction in the dipole potential with a plateau ∼35 mV (Fig. 6D).

### Cholesterol depletion in neuronal cells permits greater anesthetic-mediated dipole potential changes

**Fig. 7.**
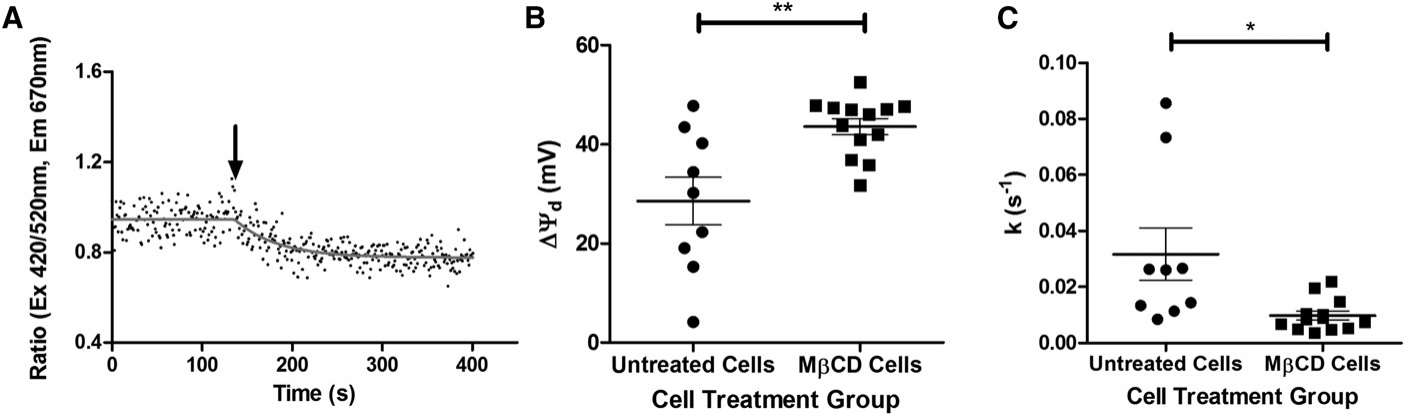
Depletion of cholesterol using MβCD in neuronal cells leads to larger changes in the dipole potential. Example experiment of ratiometric imaging (simultaneous 420/520 nm scans) of MβCD-treated cells treated with 5 mM F_3_ (A) (arrow represents the addition of F_3_). Depletion of cholesterol caused a significantly greater change in the span (B) and rate (C) of the decrease in dipole potential following 5 mM F_3_ in ratiometric titration experiments (**P = 0.0028 and *P = 0.0122, respectively).

Depletion of membrane cholesterol content by pretreatment of immortalized neuronal cells with MβCD significantly reduced their membrane dipole potential (295.6 ± 1 mV vs. 367 ± 6 mV, unpaired *t*-test, *P* < 0.0001) in agreement with previous studies (55). Subsequent application of 5 mM of F_3_ to these cells induced a large reduction in the di-8-ANEPPs fluorescence ratio (**Fig. 7A**), which equated to a significantly greater (two-tailed unpaired *t*-test, *P* = 0.0061) change in the dipole potential compared with native cells subject to exposure to the same concentration of F_3_ (Fig. 7B). The rate of F_3_-mediated dipole potential modulation in native cells was significantly greater than that found in cholesterol-depleted cells (0.032 ± 0.01 s^−1^ vs. 0.01 ± 0.002 s^−1^, two-tailed unpaired *t*-test, *P* = 0.0122) (Fig. 7C).

## DISCUSSION

This study sought to determine whether the difference in anesthetic activity of the well-described inducer/noninducer pair, **F**_3_ and **F**_6_, could be explained by their ability to differentially modulate the membrane dipole potential. Building on the work of Qin, Szabo, and Cafiso (29), who originally postulated that GA activity may be mediated by modulation of the membrane dipole potential, we sought to investigate this phenomenon using the ratiometric probe, di-8-ANEPPs, with artificial and cell membrane systems. The first attempt to provide a theory to describe the mechanism of anesthetic action was proposed independently by Meyer and Overton in 1899, who described a correlation between GA potency and oil solubility (56, 57). Since that time, several theories developed that postulated mechanisms as to how GAs may exert their effects through membrane interactions, including changes in membrane fluidity (58), volume expansion (59), or surface pressure changes (60). Although these theories provide an attractive explanation as to how GAs with such a diverse family of molecular structures could induce similar effects in vivo, over the last three decades several important criticisms have been raised against membrane-mediated theories of GA action. These include: *i*) the observation that small, but physiologically normal, changes in body temperature can induce greater changes in membrane fluidity than those achieved by GA interactions (61, 62); *ii*) the existence of nonanesthetics/nonimmobilizers, a series of molecules whose structure and lipophilicity would suggest GA activity, but do not induce anesthesia (63, 64); *iii*) an unexpected cut-off point in the positive correlation between n-alkane molecular weight and potency despite the oil partition coefficient continuing to increase (1); and *iv*) enantiomers of anesthetic molecules have varying degrees of anesthetic actions, an unexpected result given the achirality of the proposed membrane-based mechanisms of action (1).

These criticisms, combined with a seminal study by Franks and Lieb (65) that reported the affinity of GAs for the hydrophobic binding cavity in luciferase also obey the Meyer-Overton relationship, led to a shift in interest more toward protein-based theories of GA action. Since that time, a large number of predominantly protein targets for anesthetics have been identified, including activation of inhibitory ion channels, such as GABA_A_, and suppression of excitatory glutamatergic ion channels, such as NMDA receptors (66). Although a wealth of evidence now shows that GAs can modulate ion channel activity and single residue genetic modification of such channels reduces GA potency in vitro and in vivo (62, 67–69), there are few examples of a clear structure-function relationship between GAs and their ion channel targets (1). Furthermore, given the wide range of potential GA targets, a distinct lack of chemical antagonists, acquired tolerance, and high conservation of potency across the entire animal kingdom suggests that there are likely to be multiple contributing systems to GA activity, rather than a single unitary site of action (70–72). A possible solution to this problem can be found when considering that, in addition to direct ligand-protein interactions, modulation of membrane protein function can also be achieved indirectly by modulating lipid-protein interactions, particularly at the site of membrane rafts (73). For example, both GABA_A_ and NMDA receptor activity are recognized to be raft associated (74, 75) and their raft distribution is reported to change in response to activation and psychopharmacological challenge (76).

The existence of anesthetic/nonimmobilizer pairs, such as F_3_ and F_6_, with similar structures and oil partition coefficients, but very different anesthetic activity, has provided both a challenge to determining the processes underlying GA induction and an opportunity to better elucidate their mechanism of action by identifying subtle differences in behavior. The present study provides evidence to support the theory that modulation of the membrane dipole potential may contribute to the difference in anesthetic activity between the anesthetic/nonanesthetic pair, F_3_ and F_6_. This study also provides the first evidence to demonstrate GA-mediated modulation of the membrane dipole potential in a living neuronal cell.

Despite their structural similarities and lipophilic nature, F_3_ and F_6_ possess distinct average dipole moments [3.17 and 0.78 D, respectively (36)] (Fig. 1). As larger dipole moments are reported to impede the penetration of small molecules into biological membranes (77), F_3_ would be expected to localize more toward the membrane interface compared with F_6_, which would instead be found more toward the hydrophobic membrane interior. The membrane dipole potential is, itself, comprised of the electrical dipoles associated with the carbonyl group and oxygen-bonded phosphate components of the membrane surface in conjunction with the permanent molecular dipoles of water molecules occupying a restricted conformation in the membrane solvation shell at this site (78–81). The localization of F_3_ at the membrane interface would, therefore, be expected to more strongly influence the membrane dipole potential than its nonimmobilizer counterpart, F_6_.

Experimental evidence to support the difference in membrane distribution of F_3_ and F_6_ was presented in two studies by North and Cafiso (82) and Tang, Yan, and Xu (83), who used ^2^H and ^19^F NMR to report that, while F_3_ preferentially localizes to the membrane interface, F_6_ localizes to the hydrophobic core of the membrane. Interestingly, the authors also reported that halothane, isoflurane, and enflurane also localized at the membrane interface, perhaps suggesting a shared mechanism of action between these molecules. These observations were supported by subsequent studies by Tang et al. (84) and Tang, Simplaceanu, and Xu (85), who used NMR to confirm the site of the water-lipid-protein interface as the site of F_3_ interaction with gramicidin A channels. More recently, Bondarenko et al. (86) reported that using ^1^H and ^15^N solution-state NMR to show that addition of millimolar concentrations of F_3_ and isoflurane to nAChR-β2 subunit-loaded dodecyl phosphocholine micelles resulted in a change in α-helix conformation through shortening and lengthening of helix hydrogen bonds. Furthermore, the “unsaturatable” nature of some of these chemical shifts at high GA concentrations suggests a nonspecific mechanism of action. This work is in agreement with recent observations suggesting that micelles also possess a dipole potential (87) and previous work demonstrating that modulation of membrane dipole potential can induce changes in membrane peptide conformation (80). Based on this data, the difference in average dipole moment leading to the distinct membrane localization of F_3_ and F_6_ could offer an attractive mechanism to explain the difference in the ability of these molecules to modulate the membrane dipole potential.

To further test the hypothesis that modulation of membrane dipole potential may provide a potential mechanism of anesthetic action, a series of primary alcohols, which were previously reported to have anesthetic effects (51), were titrated into artificial membranes and the change in dipole potential recorded. A significant negative correlation between acyl chain length and ability to modulate the membrane dipole potential was observed. The concentration of primary alcohol required to induce a 5% change in the membrane dipole potential of PC_100%_ liposomes (∼18 mV) was found to strongly correlate with the reported EC_50_ of each molecule, including a cut-off in dipole potential modulation at C14 (tetradodecanol) (51), despite reports that primary alcohols continue to partition into lipid bilayers with increasing chain length up to 15 carbons in length (88) and no qualitative difference in the ability of the anesthetic alcohol, dodecanol, and nonanesthetic alcohol, tetradodecanol, to achieve effective levels in tadpoles, suggesting that this cut-off is not due to the reducing solubility of longer chain alcohols (89). This observation is in agreement with previous work by Ingolfsson and Anderson (90), who reported that the presence of such alcohol cut-offs can exist in the absence of a specific alcohol binding site within the system (i.e., an alcohol binding protein), supportive of indirect modulation of membrane properties as a mechanism of anesthesia induction.

Previous membrane-based explanations for the cut-off point between dodecanol and tetradodecanol include the observation by Chiou et al. (91), who, using FTIR, found that hydrogen bond breaking activity of the primary alcohol series at the membrane-water interface correlates with anesthetic potency and includes a marked cut-off point between 10 and 14 carbons. A similar hydrogen-bond breaking propensity for fluorocarbon anesthetics had previously been reported by Di Paolo and Sandorfy (92); although in both cases, the process linking interfacial hydrogen-bond breaking and anesthesia induction remained elusive. As previously described, the membrane dipole potential is comprised of the electrical dipoles associated with the carbonyl group and oxygen-bonded phosphate components of the membrane surface in conjunction with the permanent molecular dipoles of water molecules occupying a restricted conformation in the membrane solvation shell at this site (78–81). Together, these observations suggest that anesthetic-induced disruption of lipid-water hydrogen bonds at the membrane-water interface could explain the changes in the membrane dipole potential induced by F_3_ and primary alcohols up to dodecanol, but not tetradodecanol.

Although independent of the membrane dipole potential (22), modulation of membrane fluidity has been postulated as an alternative mechanism of anesthetic action (58). Using DCVJ, a small increase in membrane fluidity was observed on addition of F_3_ and F_6_ to PC_100%_ and PC_70%_Cholesterol_30%_ liposomes and the change in fluidity induced by F_3_ was found to be greater than F_6_ (although this difference was not significant). The change in fluidity was, however, found to be less than that induced by a 1°C temperature change (Table 2), in agreement with previous observations for other GA/membrane interactions (61, 62). This result strongly suggests that changes in membrane fluidity are, therefore, not responsible for GA activity, as the temperature of the mammalian brain is reported to fluctuate by up to 3°C under physiological conditions (93).

The addition of DMSO to DCVJ-labeled PC_100%_ and PC_70%_Cholesterol_30%_ liposomes (Fig. 5B, C) was found to induce a relatively large dose-dependent increase in membrane fluidity, despite DMSO possessing no anesthetic activity (94). A significantly greater change in membrane fluidity was observed on addition of DMSO to PC_70%_Cholesterol_30%_ membranes (slope: 23.0 ± 1.1% vs. 26.1 ± 0.7% Δη/1% (v/v) DMSO, extra sum-of-squares F-test, *P* = 0.0246). The marked change in membrane fluidity in response to these relatively low concentrations of DMSO may offer a novel mechanism for the recently reported toxicity of <2% concentrations of DMSO in vitro and in vivo (95), recognizing a potential link between membrane fluidity and apoptosis induction (96).

This study provides the first evidence to suggest that addition of F_3_ (but not F_6_) to immortalized neuronal cells can induce a dose-dependent reduction in membrane dipole potential equivalent to −19.1 ± 4.5 mV at 1 MAC equivalent without negatively impacting cell viability. Immortalized cell lines provide an attractive model for this work, owing to the clonal nature of the cells and the large number required for these experiments, which precluded isolation from primary tissues. A limitation of working with immortalized cells, however, is that they serve only as a model of retinal neuron cell behavior, which may not reflect the behavior of cells in vivo.

While it is extremely difficult to extrapolate clinically relevant anesthetic concentration for in vitro studies from parameters such as MAC (71), several studies have assessed the concentrations of F_3_ required to modulate the function of proteins linked to GA activity in vitro. The majority of these studies were conducted by examining the effect of GAs using two-electrode voltage clamp recording in *Xenopus* oocytes expressing membrane proteins with suspected GA activity. Low millimolar concentrations of F_3_ (typically 0.2–5 mM), but not F_6_, were reported to strongly potentiate the action of glycine on homomeric α-glycine receptor subunits in a concentration-dependent manner (97); inhibit muscarinic m_1_ receptor-induced Ca2q-dependent chloride currents (98); inhibit a number of potassium channels, including ERG-1, KCNQ2/3, and GIRK (99); and inhibit synaptosomal sodium channels (38, 100) and NMDA receptors (68). Beyond this model, Liachenko et al.(101) investigated the effects of clinically relevant concentrations (2 MAC) of F_3_ and F_6_ on K^+^-evoked glutamate and GABA release from isolated and super-fused cerebrocortical slices from mice. F_3_ (1.6 ± 0.11 mM), but not F_6_, was reported to suppress evoked glutamate release by 70%, but had no significant effects on evoked GABA release, without causing any nonspecific or irreversible changes in the brain slices. In summary, the concentrations of F_3_ reported modulating the membrane dipole potential in both artificial and neuronal membrane systems closely match those reported to modulate a range of membrane protein functions in other models.

The incorporation of 30% molar cholesterol into PC liposomes was found to reduce both the magnitude and rate of change in dipole potential on addition of F_3_. In agreement with this observation, depletion of cholesterol from neuronal cell membranes with MβCD was found to increase the change in membrane dipole potential on addition of F_3_ by almost 50%. Surprisingly, the rate of dipole potential change on depletion of membrane cholesterol was found to decrease. This may be indicative of the influence of other membrane components (i.e., charged lipids or sphingomyelin) on the interactions of F_3_ with the membrane or be a result of the recognized limitations of MβCD-mediated cholesterol depletion (102). These observations are in agreement with previous work that reported that cholesterol can inhibit the pentobarbital-mediated suppression of human brain sodium channels in planar lipid bilayers in a concentration-dependent manner (103). A possible mechanism for this process is the exclusion of F_3_ from cholesterol-containing membrane microdomains (rafts) (104), dynamic structures that may offer an additional level of control over membrane protein function. Future work will seek to isolate raft and nonraft domains from immortalized retinal neuronal cells in order to quantitatively determine the distribution of F_3_ and F_6_ between these distinct membrane domains.

A membrane dipole potential-mediated mechanism of anesthesia action can also offer an explanation why enantiomers of anesthetic molecules can possess different activities. An often overlooked, but important, property of biological membranes is that they also possess chirality. For example, PC possesses a chiral center at the position of the C2 carbon of the glycerol backbone and the plasma membrane comprises L-optical isomers of PC (105). With the growing recognition that enantioselective interactions between membrane constituents can determine the physical properties of membranes (106), including the formation and organization of membrane rafts (107), it is an intriguing hypothesis that enantiomeric anesthetics, interacting at the membrane interface at the site of the membrane dipole potential, may elicit varying degrees of activity without requiring direct protein interactions. Support for this view can be found in recent work by Bandari et al. (24), who present intriguing evidence to suggest that the membrane dipole potential is sensitive to the stereospecificity of cholesterol, which can significantly alter the dipolar field at the membrane interface that can, in turn, modulate membrane protein activity.

**Fig. 8.**
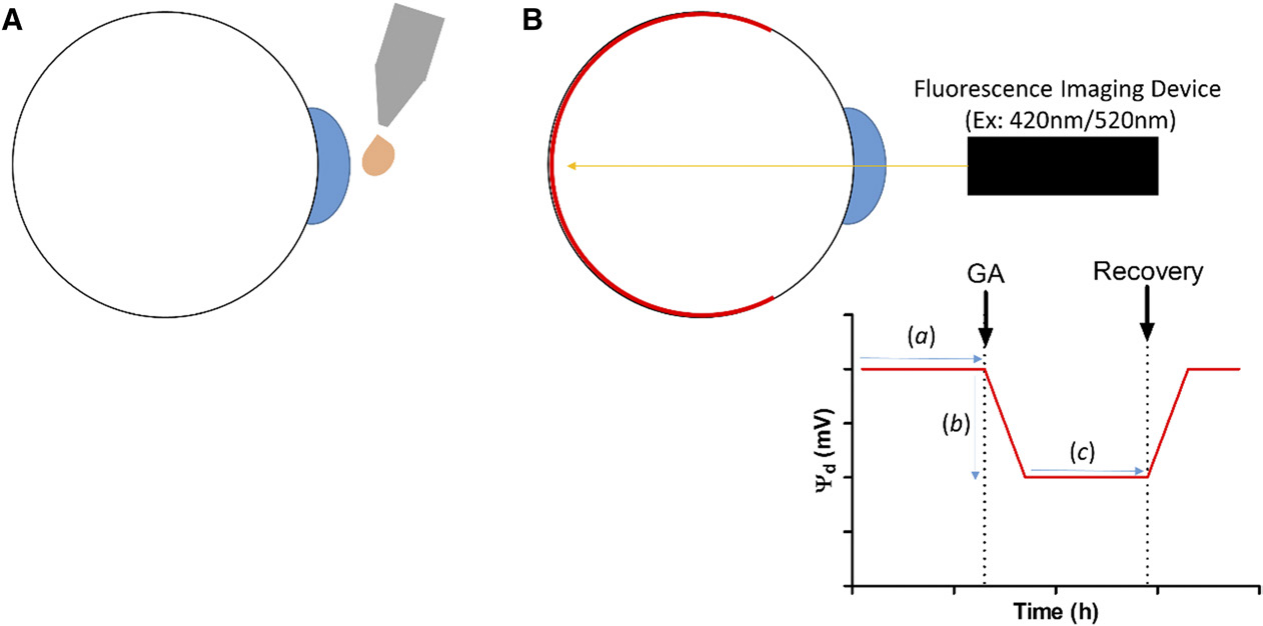
A proposed schematic of a confocal scanning laser ophthalmoscopy-based technique for monitoring GA susceptibility using di-8-ANEPPs-mediated ratiometric real-time monitoring of the membrane dipole potential. A: The di-8-ANEPPs contrast agent will be administered locally (topically) prior to surgery to permit retinal membrane dipole potential values to be recorded before administration of subclinical doses of GA. B: After GA administration, real-time depth of GA monitoring could be achieved by recording the di-8-ANEPPs fluorescent ratio; as the retina is part of the CNS, we anticipate that changes in the retinal membrane dipole potential recorded in this tissue will mirror changes reported in the brain in response to GA. These changes could present as differences in the onset of CNS dipole potential modulation after GA administration (*a*), the magnitude of the resulting dipole potential change (*b*), or a reduction in the duration of the changed dipole potential state after administration of a fixed subclinical dose of GA (*c*). Changes could manifest as a result of systemic differences in GA delivery to the CNS (i.e., changes in multidrug efflux pump activity or GA metabolism) and not necessarily simply changes in CNS membrane composition in individuals susceptible to AAGA.

Confocal scanning laser ophthalmoscopy is an established technique for noninvasive retinal visualization that is increasingly being combined with fluorescent contrast agents to increase the amount of information that can be extracted for retinal imaging purposes. For example, fluorescein and indocyanine green angiography permits diagnosis of retinal disorders via visualization of retinal and choroidal vasculature abnormalities (108) and, more recently, our group has developed the DARC (detection of apoptotic retinal cell) technology that uses fluorescently labeled annexin A5 to provide a snapshot of the number of dying cells in the retina at a specific time point (109, 110). Assuming modulation of membrane dipole potential is a common feature among other GA molecules, the ratiometric probe, di-8-ANEPPs, could provide a useful tool to monitor changes in the membrane dipole potential in response to GA induction using retinal imaging approaches. Future work will seek to establish whether modulation of membrane dipole potential can be used to predict anesthetic sensitivity for a broad range of anesthetic species and develop both a noninvasive administration technique of the di-8-ANEPPs contrast agent in rodent models and a ratiometric fluorescence-based imaging device for the real-time screening of patients’ responses to anesthesia in a preoperative environment. We anticipate that patients susceptible to AAGA will exhibit a reduced modulation in the membrane dipole potential in response to administration of subclinical doses of GAs, which could be easily monitored by noninvasive ophthalmic examination using ratiometric confocal scanning laser ophthalmoscopy, such as the device outlined in **Fig. 8**.

As AAGA may be the result of a number of factors, including failure to deliver or maintain sufficient GA in the CNS [perhaps due to differences in the activity of protein transporters or drug metabolism (111)], the authors postulate that AAGA-associated changes in GA sensitivity could manifest as: *i*) a difference in the onset of CNS dipole potential modulation after GA administration; *ii*) the magnitude of the resulting dipole potential change; or *iii*) a reduction in the duration of the changed dipole potential state after administration of a fixed subclinical dose of GA. This hypothesis assumes that there exists a strong association between the changes in membrane dipole in the CNS and GA activity in anesthetics in addition to F_3_, enflurane, isoflurane, and halothane, described previously (29), and that retinal response to GA induction mirrors changes in the rest of the CNS.

In summary, this work supports previous observations that suggest that modulation of the membrane dipole potential could provide a mechanism to explain the difference in anesthetic action of the inducer/noninducer pair, F_3_ and F_6_, and the existence of a cut-off in anesthetic activity in the primary alcohol series. An advantage of this membrane-mediated mechanism of GA action is that it may address some of the limitations of existing membrane-mediated hypotheses, including the existence of nonimmobilizers and perhaps even stereospecificity (24). Assuming that other GAs behave in a similar manner to those outlined here and in previous work (29), we anticipate that the process of dipole potential-mediated GA action will manifest via indirect modulation of membrane protein function, such as the type II interactions recently described by Richens et al. (73). We propose the use of di-8-ANEPPs-mediated reporting of the membrane dipole potential as a technique to preoperatively screen patients for anesthetic susceptibility and risk of AAGA by combining this probe with confocal scanning laser ophthalmoscopy.
